# Clinical benefit of adding oxaliplatin to standard neoadjuvant chemoradiotherapy in locally advanced rectal cancer: a meta-analysis: Oxaliplatin in neoadjuvant treatment for rectal cancer

**DOI:** 10.1186/s12885-017-3323-4

**Published:** 2017-05-12

**Authors:** Francesca De Felice, Ilaria Benevento, Anna Lisa Magnante, Daniela Musio, Nadia Bulzonetti, Rossella Caiazzo, Vincenzo Tombolini

**Affiliations:** Department of Radiotherapy, Policlinico Umberto I “Sapienza” University of Rome, Viale del Policlinico 155, 00161 Rome, Italy

**Keywords:** Locally advanced rectal cancer, Oxaliplatin, Fluoruracil, Neoadjuvant treatment, Chemoradiotherapy, Survival, Distant metastasis

## Abstract

**Background:**

Neoadjuvant fluoropirimidine (5FU)-based chemoradiotherapy (CRT) has been considered the standard of care for locally advanced rectal cancer (LARC). Whether addition of oxaliplatin (OXP) will further improve clinical outcomes is still debated. We conducted a meta-analysis to evaluate the role of OXP in this patient population.

**Methods:**

Literature searches were carried out in PubMed, Medline and Scopus databases. End points were overall survival (OS), disease free survival (DFS), local failure (LF) and distant failure (DF). Odd ratio (OR) with 95% confidence interval (CI) was calculated using random effects model.

**Results:**

Four randomized trials were included. Patients treated with OXP-5FU CRT had significantly decreased DF (OR = 0.76; 95% CI, 0.60 to 0.97; *p* = 0.03) compared to standard CRT. OS, DFS and LF were not significantly different between groups.

**Conclusions:**

OXP significantly decreased DF, but does not improve OS e DFS compared to 5FU CRT. Precise role of OXP in neoadjuvant setting of LARC remains to be determined.

## Background

The treatment of locally advanced rectal cancer (LARC) is multidisciplinary and it is developed from clinical trials evidence [1–4]. The trimodality approach, including neoadjuvant 5-fluorouracil (5FU)-based chemoradiotherapy (CRT), surgery and adjuvant chemotherapy, is considered the standard of care in this setting of patients [5]. Despite improvement in surgical techniques as well as advances in radiation therapy techniques and chemotherapeutic agents, nowadays the vast majority of recurrences in LARC are systemic [6]. Distant metastasis rate remains high (approximately 40%) and becomes the prevailing method of treatment failure.

Based on the efficacy demonstrated in colon cancer trials, the intensification regimen with oxaliplatin (OXP) as radiation-sensitizing agent in neoadjuvant CRT has been tested in several large phase III studies – STAR-01, ACCORD 12, NSAPB R-04, CAO/ARO/AIO- 04 and Chinese trial – but definitive conclusions are still pending [7–11].

Recently update of results have been presented and in light of these data we performed a meta-analysis. The aim of this meta-analysis was to evaluate whether the addition of OXP in neoadjuvant treatment for LARC could be superior to standard CRT, in term of overall survival (OS), disease free survival (DFS), local failure (LF) and distant failure (DF).

## Methods

### Selection of trials

The preferred reporting items for systematic reviews and meta-analyses (PRISMA) guidelines were followed to perform search strategy and selection processes. The meta-analysis included trials, written in English, without any restrictions on publication date. The last search was carried out on May 2016. Systematic literature electronic search was conducted in Pubmed, Medline and Scopus databases. The search term used were “radiotherapy”, “chemoradiotherapy”, “chemotherapy”, “oxaliplatin/OXP”, “fluorouracil/5FU/fluoropyrimidine”, “capecitabine/xeloda”, “rectal cancer/locally advanced rectal cancer”, “neoadjuvant”, “randomized” and “clinical trials” in the title.

To be eligible for this meta-analysis, trials needed to compare the addition of OXP to neoadjuvant CRT with standard 5FU-based CRT in LARC. To reduce publication bias, data from all clinical randomized trials, both abstract and full-text paper, were included using literature electronic databases searching (Pubmed, Medline and Scopus) and hand searching (meeting proceedings of European Society for Radiotherapy & Oncology, European Society of Medical Oncology and American Society of Clinical Oncology). Reference lists of previously published reviews and meta-analysis were explored to identify relevant citations. Trials were eligible if participants were newly diagnosed, with histologically proven adenocarcinoma of the rectum at study entry. In closer evaluation of potentially eligible articles, when two articles appeared to report results with overlapping data, only the data representing the most recent publication were included in the meta-analysis.

### Data extraction

Extracted data were recorded into standardized database according to the following parameters: first author’s surname, year of publication, trial acronym, sample size of experimental and standard group, chemotherapy regimen, drug and dosage, radiotherapy total dose and single fraction.

### Endpoints

The intent of the analysis was to evaluate disease free survival (DFS), overall survival (OS), local failure (LF) and distant failure (DF). DFS was defined as the time from the date of randomization to last follow-up, death or disease progression. OS was defined as the time from the date of randomization to last follow-up or death. LF was defined as the time from random assignment to local recurrence within the pelvis. DF was defined as the time from random assignment to distant metastasis occurrence, irrespective of whether this was a first event or not.

The number of events (death, progression and distant metastasis), when available, were derived from each study. At least one of these two outcomes should have been assessed and reported in the trial to be included in the present analysis.

### Statistical analysis

Statistical analysis was performed using Review Manager 5.0 (http://www.cochrane.org). It was based on both abstract (thus analysis of the full-text results was not performed) and full-text paper results. The analysis used odds ratio (OR) to compare results for OXP-5FU group to control patients. The pooled OR was calculated using a random-effects model. Forest plots were used for graphical representation of each study and pooled analysis. OR, variance, 95% confidence interval (CI), log [risk ratio] and standard error for each study were calculated, based on Tierney et al. method [12]. A significant two-way *p* value for comparison was defined as *p* < 0.05. Statistical heterogeneity between studies was investigated using Cochrane Q statistic (significant at *p* < 0.1) and the I^2^ value (significant heterogeneity if >50%) [13]. Publication bias was examined using Egger et al. [14] and Begg et al. [15] analyses.

## Results

### Search results

**Fig. 1:**
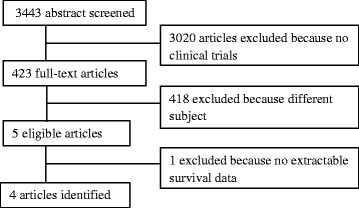
Flowchart for included and excluded trials

Four trials (3310 patients) were identified through the literature search that fulfilled the inclusion criteria (Fig. 1). Two trials were undertaken in Europe, one in USA and one in China. Baseline characteristics of these studies are outlined in Table 1. In total, 1650 patients received OXP-5FU CRT and 1660 patients were randomized to 5FU CRT. Patient characteristics, including age, gender and clinical stage disease, were well balanced between groups in all trials. In the ACCORD 12/0405-Prodige 2 trial, 509 patients were staged cT3 and 416 cases had positive lymph nodes at diagnosis. Of the 1236 patients evaluated in the German CAO/ARO/AIO-04 trial, 1071 patients were staged cT3 and 892 patients with positive lymph nodes. Baseline characteristics of patients from NSABP R-04 study were classified as stage II (T3-4 N0) in 61.7% of cases. Whereas, in the Chinese trial, a total of 127 patients had cT3 disease and the vast majority of patients (161) had positive lymph nodes.

**Table 1:** Baseline characteristics of trials

Trial			Analyzed patients		Schedule		End point	
Name	Phase	Year	Total	Per arm	Regimen	Drug and dose	primary	secondary
ACCORD12/0405-Prodige 2	III	2010	584	291	OXP-5FU CRT	Cap 1600 mg/mq daily + OXP 50 mg/mq weekly	pCR	CRM, SP, LC,PFS
				293	5FU CRT	Cap 1600 mg/mq daily		
NSAPB R-04	III	2005	1284	643	OXP-5FU CRT	5FU 225 mg/mq daily or Cap 1650 mg/mq daily + OXP 50 mg/mq weekly	LRC	OS, DFS, TLRR
				641	5FU CRT	5FU 225 mg/mq daily or Cap 1650 mg/mq daily		
CAO/ARO/AIO-04	III	2006	1236	613	OXP-5FU CRT	5FU 250 mg/mq days 1–14 and 22–35 + OXP 50 mg/mq on days 1,8,22,29	DFS	
				623	5FU CRT	5FU 1000 mg/mq days 1–5 and 29–33		
Chinese study	III	2007	206	103	OXP-5FU CRT	Cap 1600 mg/mq days 1–14 and 22–25 + OXP 60 mg/mq on days 1,8,22,29	DFS, OS	
				103	5FU CRT	Cap 1600 mg/mq days 1–14 and 22–25		

*OXP* Oxaliplatin, *5FU* 5-fluorouracil, *CRT* chemoradiotherapy, *Cap* Capecitabine, *pCR* pathologic complete response, *CRM* circumferential rectal margin, *SP* sphincter preservation, *LC* local control, *PFS* progression-free survival, *LRC* loco-regional control; *OS* overall survival, *DFS* disease-free survival, *TLRR* time to loco-regional recurrence

### Overall survival

**Fig. 2:**
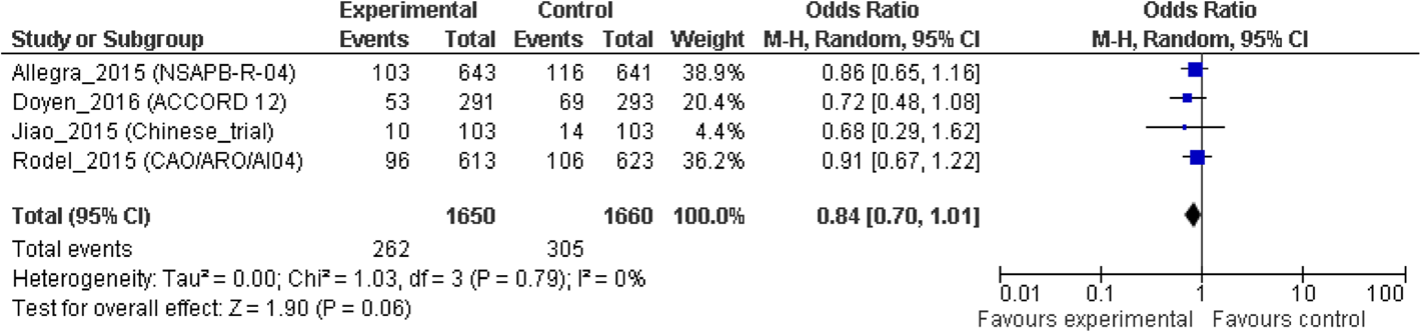
Overall survival

All trials were included in this analysis. A total of 567 deaths were recorded. No difference in OS was observed between the groups (OR = 0.84; 95% CI, 0.70 to 1.01; *p* = 0.06). No heterogeneity was observed between trials (I^2^ = 0%; χ^2^ test for heterogeneity, *p* = 0.79). Details are shown in Fig. 2.

### Disease free survival

**Fig. 3:**
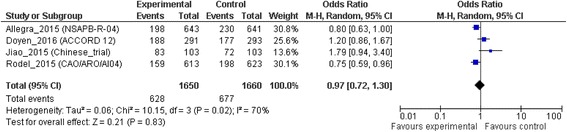
Disease free survival

The DFS analysis was based on all studies. A significant difference was not observed in favor of adding OXP to standard neoadjuvant CRT regimen (OR = 0.97; 95% CI 0.72 to 1.30; *p* = 0.83). The I^2^ value showed high heterogeneity (70%) among the studies (χ^2^ test for heterogeneity, *p* = 0.02) (Fig. 3).

### Local failure

**Fig. 4:**
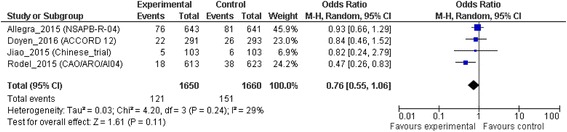
Local failure

Among the four trials analyzed, the addition of OXP was not associated with significant lower rate of local failures than 5FU alone, with an OR of 0.76 (95% CI 0.55 to 1.06; *p* = 0.11). There was not a significant heterogeneity, with an I^2^ value of 29% (χ^2^ test for heterogeneity, *p* = 0.24) (Fig. 4).

### Distant failure

**Fig. 5:**
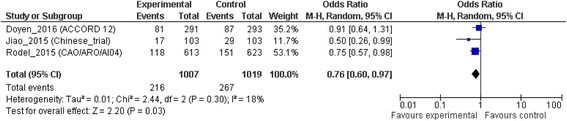
Distant failure

Data on DF were available for three trials – CAO/ARO/AIO-04 [16], ACCORD 12 [17] and Chinese Study [11] –. Thus DF analysis was conducted on a total of 2026 patients. OXP plus 5FU CRT was associated with a lower rate of distant failures than standard CRT, with an OR of 0.76 (95% CI, 0.60 to 0.97; *p* = 0.03). There was no evidence of significant statistical heterogeneity between trials (I^2^ 18%; χ^2^ test for heterogeneity, *p* = 0.30) (Fig. 5).

## Discussion

We performed a meta-analysis to compare the efficacy of neoadjuvant CRT with OXP plus 5FU to 5FU alone in LARC. The main result of this meta-analysis was that addition of OXP to standard 5FU-based CRT was related to significant clinical benefit in term of DF. Whereas, there was no significant increase in OS, as well as no lower DFS and LF rates were observed between groups, although globally a higher proportion of events was recorded in patients treated with standard CRT.

Nowadays, the treatment of LARC is multidisciplinary and it is developed from clinical trials evidence. The trimodality approach, including neoadjuvant CRT, total mesorectal excision surgery and adjuvant chemotherapy, represents the standard of care in this setting of patients, due to its value to improve local control up to 90% of cases [18]. However it does not decrease distant failures and year after year the concept of intensify treatment regimen has become progressively more common in order to improve systemic control. Based on the efficacy demonstrated in colon cancer patients [19], OXP-based intensification of neoadjuvant CRT has been tested in several randomized trials and, recently, a meta-analysis has evaluated its short term efficacy and toxicity results [20]. Briefly, OXP-5FU regimen increased pathologic complete response (OR = 1.20; 95% CI, 1.01 to 1.42) and reduced peri-operative metastasis incidence (OR = 0.51; 95% CI, 0.34 to 0.77), but increased severe toxicity rate (OR = 2.29; 95% CI, 1.31 to 4.0) compared to 5FU alone.

Given these results, the clinical outcomes analysis plays an important role in the treatment decision. Only one randomized trial demonstrated a survival benefit following addition of OXP, whereas the others failed to demonstrate it [9, 11, 16, 17]. On this background, we performed a meta-analysis to investigate the important question of identifying the optimal concomitant chemotherapy regimen to use in neoadjuvant CRT treatment in LARC. No clear evidenced resulted in prolonging OS (OR = 0.84; 95% CI, 0.70 to 1.01) and DFS (OR = 0.97; 95% CI 0.72 to 1.30) compared with the standard CRT. But OXP patients had a 24% risk reduction of developed distant metastasis (*p* = 0.03) than patients treated with single agent CRT.

There are several considerations that should be made, because the trials included in the meta-analysis did not address exactly the same end-points, and treatments were not exactly similar.

The tested primary end-points were DFS in CAO/ARO/AIO-04 trial [8], DFS and OS in Chinese trial [11], pathologic complete response in ACCORD 12 trial [7] and loco-regional control in NSAPB R-04 trial [9]. These different primary endpoints delineated the lack of statistical power to assess the chosen endpoints. Considering that the vast majority of recurrences in LARC remain systemic, the theoretical advantage of adding OXP to standard CRT should be primarily to improve distant control. Thus, a DF analysis was performed. Based on this analysis, it is possible to hypothesize that the addition of OXP is an efficient regimen to reduce the risk of systemic metastasis in LARC (OR: 0.76; 95% CI, 0.60 to 0.97). The limitations of the analysis are related to the relative limited number of trials and the heterogeneity of primary end-points. In fact, one trial had no data on distant failure [9]. Moreover it should be noted that DFS was considered the primary end-point in two out of three trials included, and it maybe has reduced the power of the analysis, but do not bias it. In addition studies were conducted in different countries and probably quality of care might have influenced the results.

Indeed these trials included different surgical approach details, such as number of examined lymph nodes and type of surgery. Total mesorectal excision was performed in the vast majority of cases. Other types of surgery, including Hartmann procedure, intersphincteric resection, transanal local excision or low anterior resection were also recorded. But the absence of complete surgical data prevents from defining a specific population that would or would not benefit from OXP-5FU regimen. Thus whether the inclusion of OXP into 5FU-based CRT could reflect the new treatment option for patients with LARC cannot be resolved by our meta-analysis. Surely, the benefit of adding OXP on DF could justify the proven substantial increase in acute toxicity, especially severe diarrhea [20]. Among the trials included, patients received substantially similar OXP regimens – 50 mg/m^2^ once a week or 60 mg/m^2^ on days 1, 8, 22, 29 – but heterogeneous 5FU schedule. In CAO/ARO/AIO-04 trial [8], the 5FU schedules even differed in the two treatment groups – 250 mg/m^2^ daily in experimental group versus 1000 mg/m^2^ weeks 1 and 5 of RT in control group –, whereas the other trials used the same 5FU regimen for both treatment – 225 mg/m^2^ daily or 1600 mg/m^2^ daily – [7, 9, 11]. Considering that 5FU is probably the main responsible for gastrointestinal toxicity, including diarrhea, nausea and vomiting, we believe that acute toxicity could be easily minimize by decrease the individual 5FU doses (200 mg/m^2^) and increase the frequency of administration of weekly OXP (to a maximal total dose of 300 mg), maintaining therapeutic effectiveness [21]. In our published retrospective analysis, we found a considerable decrease in toxicity (17%), particularly in terms of severe diarrhea (4%) compared to grade 3/4 toxicity rate reported in randomized studies, ranged from 21.4% to 41.9% [7–11].

To our knowledge, this is the first meta-analysis with long-term follow-up data to show a DF benefit of adding OXP to 5FU-based CRT when compared with standard treatment with 5FU alone for patients with LARC. However results need to be interpreted with caution, because it is an abstract-based meta-analysis. Although abstract included statistical quality details, definitive full-text results could improve data analysis precision and produce more robust conclusions. In particular, subgroup analysis might reveal different results for some patients, such as node positive versus node negative patients. However, reduction in DF could be considered a sensible result, reflecting itself a relevant benefit for patients, regardless of survival improvement.

## Conclusions

Adding OXP to standard CRT had significant effect on distant metastasis control, however no survival benefit was derived. Further data are needed to clarify the precise role of OXP in neoadjuvant setting of LARC.

## Abbreviations

5FU: Fluoropirimidine
CI: Confidence interval
CRT: Chemoradiotherapy
DF: Distant failure
DFS: Disease free survival
LARC: Locally advanced rectal cancer
LF: Local failure
OR: Odd ratio
OS: Overall survival
OXP: Oxaliplatin

## References

[1] Schmoll HJ, Van Cutsem E, Stein A, Valentini V, Glimelius B, Haustermans K, Nordlinger B, van de Velde CJ, Balmana J, Regula J, Nagtegaal ID, Beets-Tan RG, Arnold D, Ciardiello F, Hoff P, Kerr D, Köhne CH, Labianca R, Price T, Scheithauer W, Sobrero A, Tabernero J, Aderka D, Barroso S, Bodoky G, Douillard JY, El Ghazaly H, Gallardo J, Garin A, Glynne-Jones R, Jordan K, Meshcheryakov A, Papamichail D, Pfeiffer P, Souglakos I, Turhal S, Cervantes A. ESMO Consensus Guidelines for management of patients with colon and rectal cancer. a personalized approach to clinical decision making. Ann Oncol. 2012;23(10):2479–516. 10.1093/annonc/mds236 23012255

[2] Sauer R, Becker H, Hohenberger W, Rödel C, Wittekind C, Fietkau R, Martus P, Tschmelitsch J, Hager E, Hess CF, Karstens JH, Liersch T, Schmidberger H (2004). Raab R; German rectal cancer study group. Preoperative versus postoperative chemoradiotherapy for rectal cancer. N Engl J Med.

[3] Bosset JF, Collette L, Calais G, Mineur L, Maingon P, Radosevic-Jelic L, Daban A, Bardet E, Beny A (2006). Ollier JC; EORTC radiotherapy group trial 22921. Chemotherapy with preoperative radiotherapy in rectal cancer. N Engl J Med.

[4] Sainato A (2014). Cernusco Luna Nunzia V, Valentini V, De Paoli A, Maurizi ER, Lupattelli M, Aristei C, Vidali C, Conti M, Galardi A, Ponticelli P, Friso ML, Iannone T, Osti FM, Manfredi B, Coppola M, Orlandini C, Cionini L. no benefit of adjuvant fluorouracil Leucovorin chemotherapy after neoadjuvant chemoradiotherapy in locally advanced cancer of the rectum (LARC): long term results of a randomized trial (I-CNR-RT). Radiother Oncol.

[5] National Comprehensive Cancer Network Guidelines, Rectal Cancer, Version 2.2016. www.nccn.org

[6] Weiser MR, Zhang Z, Schrag D. Locally advanced rectal cancer: time for precision therapeutics. Am Soc Clin Oncol Educ Book. 2015:e192–6. 10.14694/EdBook_AM.2015.35.e192 25993172

[7] Gérard JP, Azria D, Gourgou-Bourgade S, Martel-Laffay I, Hennequin C, Etienne PL, Vendrely V, François E, de La Roche G, Bouché O, Mirabel X, Denis B, Mineur L, Berdah JF, Mahé MA, Bécouarn Y, Dupuis O, Lledo G, Montoto- Grillot C, Conroy T. Comparison of two neoadjuvant chemo- radiotherapy regimens for locally advanced rectal cancer: results of the phase III trial ACCORD 12/0405-Prodige 2. J Clin Oncol 2010; 28: 1638–1644. 10.1200/JCO.2009.25.8376 20194850

[8] Rödel C, Liersch T, Becker H, Fietkau R, Hohenberger W, Hothorn T, Graeven U, Arnold D, Lang-Welzenbach M, Raab HR, Sülberg H, Wittekind C, Potapov S, Staib L, Hess C, Weigang-Köhler K, Grabenbauer GG, Hoffmanns H, Linde-mann F, Schlenska-Lange A, Folprecht G, Sauer R. Preopera- tive chemoradiotherapy and postoperative chemotherapy with fluorouracil and oxaliplatin versus fluorouracil alone in locally advanced rectal cancer: initial results of the German CAO/ARO/AIO-04 randomised phase 3 trial. Lancet Oncol 2012; 13: 679–687. 10.1016/S1470-2045(12)70187-0 22627104

[9] O'Connell MJ, Colangelo LH, Beart RW, Petrelli NJ, Allegra CJ, Sharif S, Pitot HC, Shields AF, Landry JC, Ryan DP, Parda DS, Mohiuddin M, Arora A, Evans LS, Bahary N, Soori GS, Eakle J, Robertson JM, Moore DF Jr, Mullane MR, Marchello BT, Ward PJ, Wozniak TF, Roh MS, Yothers G, Wolmark N. Capecitabine and oxaliplatin in the preoperative multimodality treatment of rectal cancer: surgical end points from National Surgical Adjuvant Breast and Bowel Project trial R-04. J Clin Oncol. 2014;32(18):1927–34. 10.1200/JCO.2013.53.7753 PMC4050205 24799484

[10] Aschele C, Cionini L, Lonardi S, Pinto C, Cordio S, Rosati G, Artale S, Tagliagambe A, Ambrosini G, Rosetti P, Bonetti A, Negru ME, Tronconi MC, Luppi G, Silvano G, Corsi DC, Bo- chicchio AM, Chiaulon G, Gallo M, Boni L. Primary tumor response to preoperative chemoradiation with or without oxaliplatin in locally advanced rectal cancer: pathologic results of the STAR-01 randomized phase III trial. J Clin Oncol 2011; 29: 2773–2780. 10.1200/JCO.2010.34.4911 21606427

[11] Jiao D, Zhang R, Gong Z, Liu F, Chen Y, Yu Q, Sun L, Duan H, Zhu S, Liu F, Wang J, Jia J (2015). Fluorouracil-based preoperative chemoradiotherapy with or without oxaliplatin for stage II/III rectal cancer: a 3-year follow-up study. Chin J Cancer Res.

[12] Tierney JF, Stewart LA, Ghersi D, Burdett S, Sydes MR (2007). Practical methods for incorporating summary time-to-event data into meta-analysis. Trials.

[13] Higgins JPT, Thompson SG, Deeks JJ, Altman DG (2003). Measuring inconsistency in meta-analyses. BMJ.

[14] Egger M, Davey SG, Schneider M, Minder C (1997). Bias in meta- analysis detected by a simple, graphical test. BJM.

[15] Begg CB, Mazumdar M (1994). Operating characteristics of a rank correlation test for publication bias. Biometrics.

[16] Rödel C, Graeven U, Fietkau R, Hohenberger W, Hothorn T, Arnold D, Hofheinz RD, Ghadimi M, Wolff HA, Lang-Welzenbach M, Raab HR, Wittekind C, Ströbel P, Staib L, Wilhelm M, Grabenbauer GG, Hoffmanns H, Lindemann F, Schlenska-Lange A, Folprecht G, Sauer R, Liersch T, German Rectal Cancer Study Group. Oxaliplatin added to fluorouracil-based preoperative chemoradiotherapy and postoperative chemotherapy of locally advanced rectal cancer (the German CAO/ARO/AIO-04 study): final results of the multicentre, open-label, randomised, phase 3 trial. Lancet Oncol. 2015;16(8):979–89. 10.1016/S1470-2045(15)00159-X 26189067

[17] Francois E, Gourgou-Bourgade S, Azria D, Conroy T, Bouche O, Doyen J, Seitz JF, Mineur L, Etienne PL, Gerard JP. ACCORD12/0405-Prodige 2 phase III trial neoadjuvant treatment in rectal cancer: Results after 5 years of follow-up. J Clin Oncol 34, 2016 (suppl 4S; abstr 490).

[18] Valentini V, Beets-Tan R, Borras JM, Krivokapić Z, Leer JW, Påhlman L, Rödel C, Schmoll HJ, Scott N, Velde CV, Verfaillie C (2008). Evidence and research in rectal cancer. Radiother Oncol.

[19] Sanoff HK, Carpenter WR, Martin CF, Sargent DJ, Meyerhardt JA, Stürmer T, Fine JP, Weeks J, Niland J, Kahn KL, Schymura MJ, Schrag D (2012). Comparative effectiveness of oxaliplatin vs non-oxaliplatin-containing adjuvant chemotherapy for stage III colon cancer. J Natl Cancer Inst.

[20] An X, Lin X, Wang FH, Goodman K, Cai PQ, Kong LH, Fang YJ, Gao YH, Lin JZ, Wan DS, Pan ZZ, Ding PR (2013). Short term results of neoadjuvant chemoradiotherapy with fluoropyrimidine alone or in combination with oxaliplatin in locally advanced rectal cancer: a meta analysis. Eur J Cancer.

[21] De Felice F, Musio D, Magnante AL, Bulzonetti N, Benevento I, Caiazzo R, Tombolini V. Disease Control, Survival, and Toxicity Outcome After Intensified Neoadjuvant Chemoradiotherapy for Locally Advanced Rectal Cancer: A Single-Institution Experience. Clin Colorectal Cancer. 2016. pii: S1533–0028(16)30018–4. 10.1016/j.clcc.2016.02.006 26952656

